# The development and use of data warehousing in clinical settings: a scoping review

**DOI:** 10.3389/fdgth.2025.1599514

**Published:** 2025-06-05

**Authors:** Shiyang Lyu, Simon Craig, Gerard O'Reilly, David Taniar

**Affiliations:** ^1^ Software Systems and Cybersecurity Department, Faculty of Information Technology, Monash University, Melbourne, VIC, Australia; ^2^ Paediatric Emergency Department, Monash Medical Centre, Monash Health, Melbourne, VIC, Australia; ^3^ Department of Paediatrics, School of Clinical Sciences, Faculty of Medicine, Nursing and Health Sciences, Monash University, Melbourne, VIC, Australia; ^4^ Emergency and Trauma Centre, Alfred Health, Melbourne, VIC, Australia; ^5^ School of Public Health and Preventive Medicine, Faculty of Medicine, Nursing and Health Sciences, Monash University, Melbourne, VIC, Australia

**Keywords:** healthcare, data warehouse, digital health, hospital data systems, clinical systems

## Abstract

**Introduction:**

The emergence of data warehousing in clinical settings has greatly enhanced data analysis capabilities, facilitating the accurate and comprehensive extraction of valuable information. This scoping review explores the contributions of data warehouses in clinical settings by analysing the strengths, challenges and implications of each type of data warehouse, with a particular focus on general and specialised types.

**Methods:**

This scoping review adheres to the Preferred Reporting Items for Systematic Reviews and Meta-Analyses (PRISMA) guidelines. We searched four databases (PubMed, CINAHL, Scopus and IEEE-Xplore), identifying peer-reviewed, English-language studies from 1st January 2014 to 1st January 2024, that focus on data warehousing in healthcare, covering either general or specialised data warehouse applications. Python programming was used to extract the search results and transform the data into a tabular format for analysis.

**Results:**

After removing 1,194 duplicates, 4,864 unique papers remained. Abstract screening excluded 4,590 as irrelevant, leaving 274 for full-text evaluation. In total, 27 papers met the inclusion criteria, of which 17 focused on general data warehouses and 10 on specialised data warehouses.

General data warehouses were found to be primarily used to address data integration issues, particularly for electronic health record (EHR)/ Electronic medical Record (EMR) and general clinical data. These warehouses typically use a star schema architecture with online analytical processing (OLAP) and query analysis capabilities. In contrast, specialised data warehouses were focused on improving the quality of decision support by handling a wide range of data specific to diseases, using specialised architectures and advanced artificial intelligence (AI) capabilities to address the unique and complex challenges associated with these tasks.

**Conclusions:**

General purpose data warehouses effectively integrate disparate data sources to provide a comprehensive view of disease management, patient care, and resource management. However, their flexibility and analytical capabilities need improvement. In contrast, specialised data warehouses are gaining popularity for their focus on specific diseases or research purposes, using advanced tools such as data mining and AI for superior analytical performance. Despite their innovative designs, these specialised warehouses face scalability challenges due to their customised nature. Addressing these challenges with advanced analytics and flexible architectures is critical.

## 1 Introduction

The rapid digitization of healthcare has generated an increasing volume of clinical data, leading to a growing interest in technologies that can manage, integrate, and analyse clinical information to improve patient care and operational efficiency. A clinical data warehouse (CDW) is a centralized repository designed to collect, store, and process data from multiple healthcare information systems, such as electronic health records (EHRs), laboratory systems and radiology systems (1). It plays a key role in the management and analysis of vast amounts of digital health data in the context of clinical and healthcare industries. As digital health technologies continue to advance, the implementation of data warehouses has become increasingly critical for improving clinical workflows, patient care, and research capabilities. The healthcare industry has seen a growing adoption of data warehouses, driven by the need to use data to improve various aspects of clinical practice and administration. Healthcare providers and institutions, including hospitals, clinics, and research facilities, rely on data warehouses to integrate and analyse disparate data sources, providing comprehensive insights that support strategic planning (2).

Previous research has shown that clinical data warehouses can effectively integrate disparate data sources to provide a comprehensive view that supports both clinical and operational decisions by integrating EHR, laboratory systems, and other clinical databases into a single repository (1, 3, 4). In addition, clinical data warehouses support research by providing rich datasets for secondary use, allowing the extraction and analysis of diverse patient data to address broader clinical or specific diseases (2), such as acute kidney injury (5) and cancer management (6, 7).

However, inefficiencies and challenges associated with clinical data warehouses, such as several data quality issues, particularly inconsistencies and inaccuracies in data collected from different sources (1, 2, 7). These data quality issues can significantly reduce the effectiveness of data warehouses, making it difficult for healthcare providers to trust the insights generated. Integrating data from disparate systems often involves complex Extract, Transform, Load (ETL) processes, which can be resource-intensive and error-prone (5, 8). These challenges can limit the scalability and sustainability of data warehouse initiatives. At the same time, the complexity of clinical environments, the diversity of data, and growing analytics needs have led to the development of different types of data warehouses.

Despite the growing importance of data in clinical settings, there is a lack of comprehensive understanding of the specific benefits and limitations of data warehouses in this context. The rationale for this scoping review is fourfold: (1) to understand the use of data warehouses in clinical settings, (2) to explore the effectiveness of data warehouses in clinical environments, (3) to identify the characteristics and components of data warehouses, and (4) to learn the benefits and limitations associated with the use of data warehouses in clinical practice.

Therefore, the aim of this scoping review is to comprehensively explore the contributions of data warehouses in clinical settings by analysing the strengths, challenges and implications of each type of data warehouse and to provide a comprehensive understanding of their respective roles in healthcare. More specifically, this scoping review aims to address the identified research gap by focusing on two main types of data warehouse: (1) *General data warehousing* is designed to improve the overall data analysis capabilities of the entire healthcare organisation by integrating disparate data sources to provide a holistic view of hospital workflows, patient care and resource management (1, 2, 7). (2) *Specialised data warehousing* is designed to meet specific clinical or research needs, using targeted data integration and advanced analytic tools to address the specific needs of particular diseases or research purposes (5).

The components of this scoping review include: (1) the population, which comprises data warehouses used in clinical settings; (2) the concepts examined, include the types and sources of data involved, the data warehouse architecture, the analytics technology used, and post-implementation challenges; and (3) the context of this review, which covers clinical settings, including healthcare organizations, clinical institutions and hospitals that have implemented data warehouses to support decision making.

## 2 Material and method

This scoping review study adheres to the established Preferred Reporting Items for Systematic Reviews and Meta-Analyses (PRISMA) guidelines (9), as detailed in the Supplementary Table S1.

### 2.1 Information sources and search strategy

Four major electronic bibliographic databases were selected for the literature search: PubMed, CINAHL, Scopus and IEEE-Xplore. Of the four databases, PubMed and CINAHL specialise in the health and clinical fields. IEEE-Xplore, on the other hand, focuses on technology and engineering research, providing insights into the technical aspects of data warehousing. Lastly, we utilised Scopus, a general and multidisciplinary database, to ensure that different perspectives on data warehousing were captured. This combination of databases ensures a thorough and balanced review of both clinical and technical literature.

A novel keyword search strategy was developed to effectively identify relevant studies given the interdisciplinary nature of this scoping review. This strategy involved categorising keywords into two main domains: the data warehouse technology domains and the clinical domain. The technical dimension of the review aims to address critical components of data warehousing such as data sources and structure, architecture, and analytic capability. These elements are essential to understand the infrastructure and capabilities of healthcare data warehouses. Then, from the clinical perspective, the review focuses on how data warehouses are used in different healthcare scenarios. This includes applying data warehousing technology to manage different diseases, improving hospital administrative and clinical processes, and adapting to the unique healthcare challenges of different countries. Each selected study is analysed to determine how data warehouses meet specific clinical needs and contribute to improved health outcomes. Only those papers falling into both domains were to be included. The complete search strategy can be found in Supplementary Table S2.

### 2.2 Inclusion and exclusion criteria

Two reviewers jointly developed the eligibility criteria for this scoping review. The inclusion criteria of this scoping review are: (1) Studies that focus on the use of data warehousing in the clinical or healthcare setting (2) Studies that specifically address the implementation, data sources, ETL, architecture, analytical capabilities, or current limitations in the healthcare context of clinical applications. (3) Studies that address either general data warehouse implementations that aim to improve the overall data analysis capabilities, or specialized data warehouses that are designed to meet specific clinical or research needs. (4) Studies that have been published within the last ten years, between 1 January 2014 and 1 January 2024, ensuring that the review focuses on recent developments and current trends in the field, capturing the latest advances and innovations while providing a contemporary snapshot of the research landscape. (5) Studies which were peer-reviewed and English-language articles, ensuring that the included studies met a standard of academic quality and credibility.

The exclusion criteria are: (1) Studies that do not focus on the use of data warehouses in clinical settings; (2) Statistical or modelling studies that merely mention the data warehouse as a source of data without focusing on its implementation or impact; (3) Studies that fall out of the published range, non-English publications, or non-peer-reviewed studies.

### 2.3 Study selection and data extraction

The three main steps in the study selection process: identification, screening and eligibility assessment, were developed by two reviewers. The Python programming language was utilized during this process, which was tested on the first ten papers to ensure consistency and accuracy. In the identification step, studies with identical DOI numbers and article titles were considered duplicates and removed from the study dataset. Then, in the screening phase, the titles and abstracts of the identified studies were used to ascertain their relevance to the specified keyword strategy. At the eligibility stage, the full texts of the selected studies were assessed independently by two reviewers to ensure that they met the inclusion criteria and were relevant to both the data warehouse and clinical domains.

In data extraction form, the following methodological and outcome variables were collected from each study by two reviewers. Overall, the form included:

• General information: This included authors, publication year, paper title, abstract and author/publication keywords.
• Data warehouses domain: This included the data warehouse technology used, the type of data used, the type of data warehouse architecture, and the intended use of the data warehouse.
• Clinical domains: This included the type of diseases involved, the area of implementation and the core focus area.

A detailed description of the data extraction form is attached in Supplementary Table S3. Any disagreements between the reviewers were resolved by discussion. Moreover, the limited functionality of the PubMed database prevents direct extraction of abstracts and author/publication keywords from search results. The Python package *Pymed* has been developed as a solution to this problem and facilitates the retrieval of medical literature from PubMed (10). *Pymed* provides a simple interface for querying and extracting records, including keywords and abstracts, from PubMed. For the CINAHL database, the results obtained were initially stored in a text file. Python programming was used to extract the attributes of each article and convert the results into a tabular format. In total, data from all four databases were stored in a tabular format for analysis, allowing for efficient processing and comparison.

### 2.4 Data synthesis

A structured synthesis was conducted based on the data extracted from each study using a data extraction form. The purpose of the synthesis was to identify key themes, patterns, and differences among the included studies. All data warehouses were categorized into two types for ease of comparison: data warehouses and databases: General Data Warehousing, which aims to improve overall general data analysis capabilities, and Specialized Data Warehousing, which targets specific clinical conditions or research objectives.

A comparative analysis was then conducted to identify how the two categories differed. Six thematic areas were used to guide this analysis: (1) core focus areas, (2) data sources, (3) analytic capabilities, (4) data structure (5) data transformation process (6) data warehouse architecture, and (7) post-implementation challenges. This comparison helped to understand the unique strengths and challenges associated with each type of data warehouse. To visually present findings, sankey diagrams were used for perspectives involving proportional relationships-specifically, the distribution of focus areas and data sources across both warehouse types. For technical aspects such as data structures, transformation processes, and architecture as well as post-implementation challenges, summary tables were used to present detailed information at the study level.

## 3 Results

**Figure 1:**
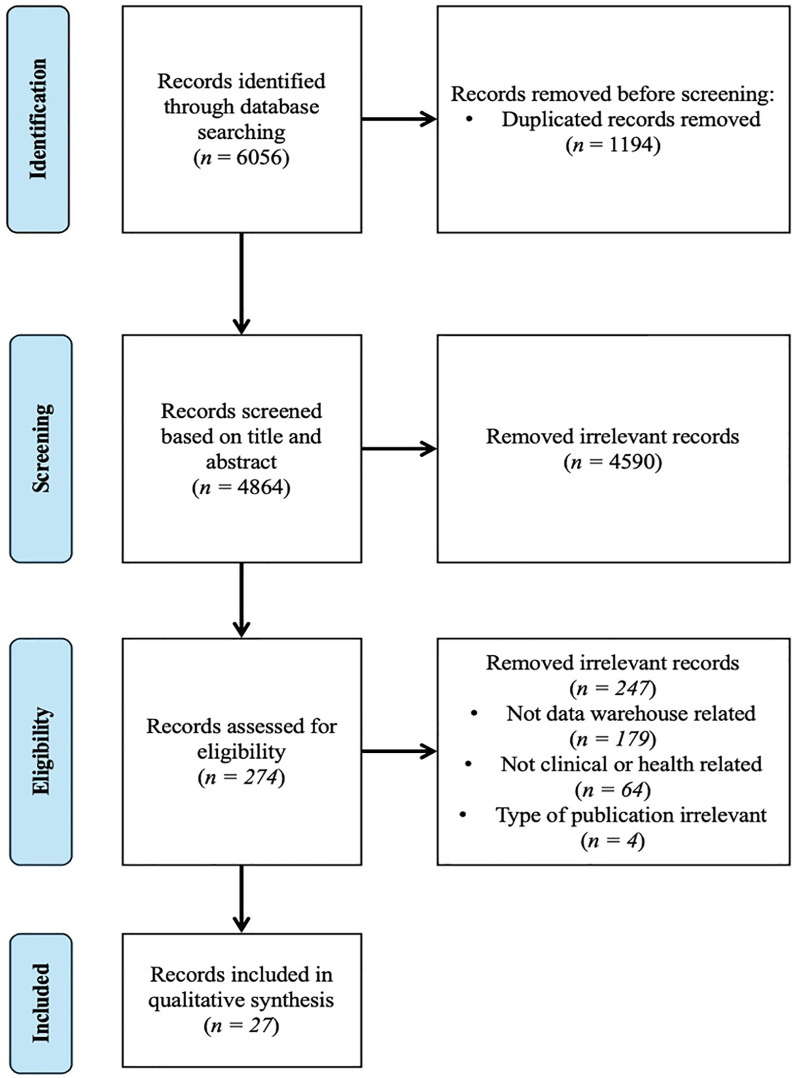
PRISMA flowchart of data warehouse paper selection in clinical and health research. The diagram illustrates the number of records identified from the database (PubMed, Scopus, IEEE and CINAHL), the exclusion process and the inclusion process.

Figure 1 presents the PRISMA flow diagram. The preliminary search yielded 6,056 papers from PubMed, Scopus, IEEE Xplore, and CINAHL. Following the removal of 1,194 duplicate papers, a total of 4,864 unique papers were identified. A total of 4,590 papers were excluded from the review based on abstract screening, as they were deemed irrelevant to the scope of the study. A total of 274 papers were deemed eligible for full-text assessment. Of these, only 27 met the criteria for inclusion in the final review and were included in the study. Table 1 presents a detailed summary of characteristics of each selected study.

**Table 1:** Characteristics of 27 selected studies included in the scoping review.

Author, Year, [Ref]	Brief	Data sources	Type of data warehouse identified	Number of hospitals involved	Core focus area	Post-implementation challenges
Priou et al. 2023, (1)	Analysis of whether the CDW can deliver on its promise, based on expert interviews	Electronic Health Record (EHR)	General Data warehouse	Not specified	Complexities of Data Integration	Hospital subsystem integration
Henley-Smith et al. 2019, (2)	Proposed framework for the quality of data in the CDW for secondary use	EHR	General Data warehouse	Not specified	Issues in Data Quality Management	Inconsistent use, incorrect coding due to different EHR systems
Wade et al. 2014, (3)	Proposed dimensional bus model to integrate EMR, sponsored study and biorepository data.	Electronic medical Record (EMR)	General Data warehouse	More than 5 Hospitals/Clinics	Challenges in Decision Support Quality	Improve query formulation and execution time
Sebaa et al. 2017, (4)	Developed a decision support system for medical resource allocation in Bejaia.	General clinical or health data	General Data warehouse	More than 5 Hospitals/Clinics	Concerns in Data Privacy and Security	Data protection and privacy policies in the different regions
Baghal et al. 2019, (5)	Proposed a graph model for visualizing and exploring acute kidney injury (AKI) entities.	EMR: Acute kidney injury (AKI) related	Specialised data warehouse (Image based data)	One Hospital only	Challenges in Decision Support Quality	The traditional relational model lacks agility and scalability for evolving data needs.
Atay et al. 2020, (6)	Development of a clinical data warehouse for lung cancer data	Prostate, lung, colorectal, and ovarian (PLCO) data	Specialised data warehouse	United States National Cancer Institute	Challenges in Decision Support Quality	Effective decision-making remains challenging.
Ritzwoller et al. 2014, (7)	Assessing the accuracy of treatment data in the Cancer Research Network's Virtual Data Warehouse (VDW)	Tumor Registry data	Specialised data warehouse	More than 5 Hospitals/Clinics	Issues in Data Quality Management	Lack of published validation studies assessing the quality of automated data
De Assis Vilela et al. 2023, (8)	Suggested real-time extract, transform and load (ETL) into the data warehouse	General clinical or health data	General Data warehouse	Not specified	Complexities of Data Integration	Dealing with data collection frequency
Freund et al. 2014, (11)	Use data warehouse technology to characterise medication use across age groups.	EMR	General Data warehouse	More than 5 Hospitals/Clinics	Concerns in Data Privacy and Security	Limited support for medical resource allocation.
Puppala et al. 2016, (12)	A focus on the issue of data protection and data security in the CDW	EHR	General Data warehouse	One Hospital only	Concerns in Data Privacy and Security	Data privacy concern
Nobles et al. 2015, (13)	EHR data quality assessment for secondary analysis	EHR	General Data warehouse	More than 5 Hospitals	Issues in Data Quality Management	
Neamah, 2020, (14)	Integration of different sources of EHR data	EHR	General Data warehouse	Not specified	Challenges in Decision Support Quality	Data integration
Krause et al. 2015, (15)	Facilitate health planning by building and coordinating infrastructure, capacity, tools, and resources.	General clinical or health data	General Data warehouse	More than 5 Hospitals/Clinics	Complexities of Data Integration	Data has historically been held in data silos and not easily shared.
Khan et al. 2015, (16)	Explores health data warehousing and mining in Bangladesh.	General clinical or health data	General Data warehouse	More than 5 Hospitals/Clinics	Complexities of Data Integration	Unstructured Data Integration Issue
McGlothlin et al., 2016, (17)	Using an enterprise data warehouse and business intelligence tools to improve clinical outcomes	EHR	General Data warehouse	One Hospital only	Challenges in Decision Support Quality	
Wood et al. 2016, (23)	Building a data warehouse of patients undergoing Roux-en-Y gastric bypass (RYGB) surgery	Obesity related EHR	Specialised data warehouse (Obesity)	Geisinger Bariatric Surgery clinical program	Complexities of Data Integration	The availability and integrity of different types of data in EHRs can vary widely.
Abouzahra et al. 2014, (18)	Framework for Integrating EHR Data to Enhance Clinical Decision-Making	EHR	General Data warehouse	More than 5 Hospitals/Clinics	Complexities of Data Integration	EHR Interoperability and Information Overload Challenges
Baghal, 2019, (24)	Enhances DW analysis using NLP for pathology documents.	EMR: Clinical reports, Pathology reports	Specialised data warehouse (Text based data)	Not specified	Challenges in Decision Support Quality	
Teixeira et al. 2015, (25)	Proposed DW for medical image management	Image data, such as brain images	Specialised data warehouse (Image based data)	Not specified	Challenges in Decision Support Quality	
Shin et al. 2014, (19)	Characterise the issue of CDW implementation	General clinical or health data	General Data warehouse	More than 5 Hospitals/Clinics	Challenges in Decision Support Quality	Efficient CDW implementation remain undefined.
Artemova et al. 2021, (26)	Established a COVID-19 data warehouse for daily spatial-temporal monitoring.	COVID-19 data	Specialised data warehouses	One Hospital only	Challenges in Decision Support Quality	Addressing challenges for hospitals and city administrations.
Agapito et al. 2020, (27)	Proposed a COVID-19 Data WAREHOUSE	COVID-19 data	Specialised data warehouse	Not specified	Challenges in Decision Support Quality	Addressing COVID-19 data integration issues.
Scheer et al. 2020, (28)	Proposed a new method for monitoring ICU transfers using a data warehouse.	Intensive care units (ICUs) related data	Specialised data warehouse	More than 5 Hospitals/Clinics	Challenges in Decision Support Quality	Intensive care units (ICUs) are under constant pressure to balance capacity
Faridoon et al. 2022, (20)	Proposed an advanced data privacy management architecture	General clinical or health data	General Data warehouse	Not specified	Concerns in Data Privacy and Security	Handling sensitive health data
Ren et al. 2018, (21)	Proposed an ontology-based method for medical data warehouse to prevent semantic heterogeneity.	EMR	General Data warehouse	Not specified	Complexities of Data Integration	Medical data sources face diverse types, large volumes, and lack associations.
Karami et al. 2017, (22)	Established CDW to reduce the incidence of disease, improve patient care	General clinical or health data	General Data warehouse	Not specified	Challenges in Decision Support Quality	Evaluate disease management programs affecting patient quality of life.
Grammatico-Guillon et al. 2019, (29)	Evaluates data warehouse use for paediatric antibiotic prescribing patterns.	Antibiotic Prescribing Data	Specialised data warehouse	One Hospital only	Challenges in Decision Support Quality	

Summary of data sources, data warehouse types, number of hospitals involved, and types of challenges addressed.

Based on the results of the synthesis, the two main applications of clinical data warehousing identified in our review of 27 papers were:

(1) *General data warehousing*: improving overall data analysis capabilities across the hospital, appearing in 17 of the 27 papers reviewed (1–4, 8, 11–22).
(2) *Specialized data warehousing* aimed at improving disease-specific data analysis or supporting research-oriented goals, appearing in 10 of the 27 papers reviewed (5–7, 23–29)

The following subsections summarize two main applications of clinical data warehousing from 7 key perspectives.

### 3.1 Functional aspects of clinical data warehousing: core focus areas, data sources, and analytical capabilities

#### 3.1.1 Core focus areas

**Figure 2:**
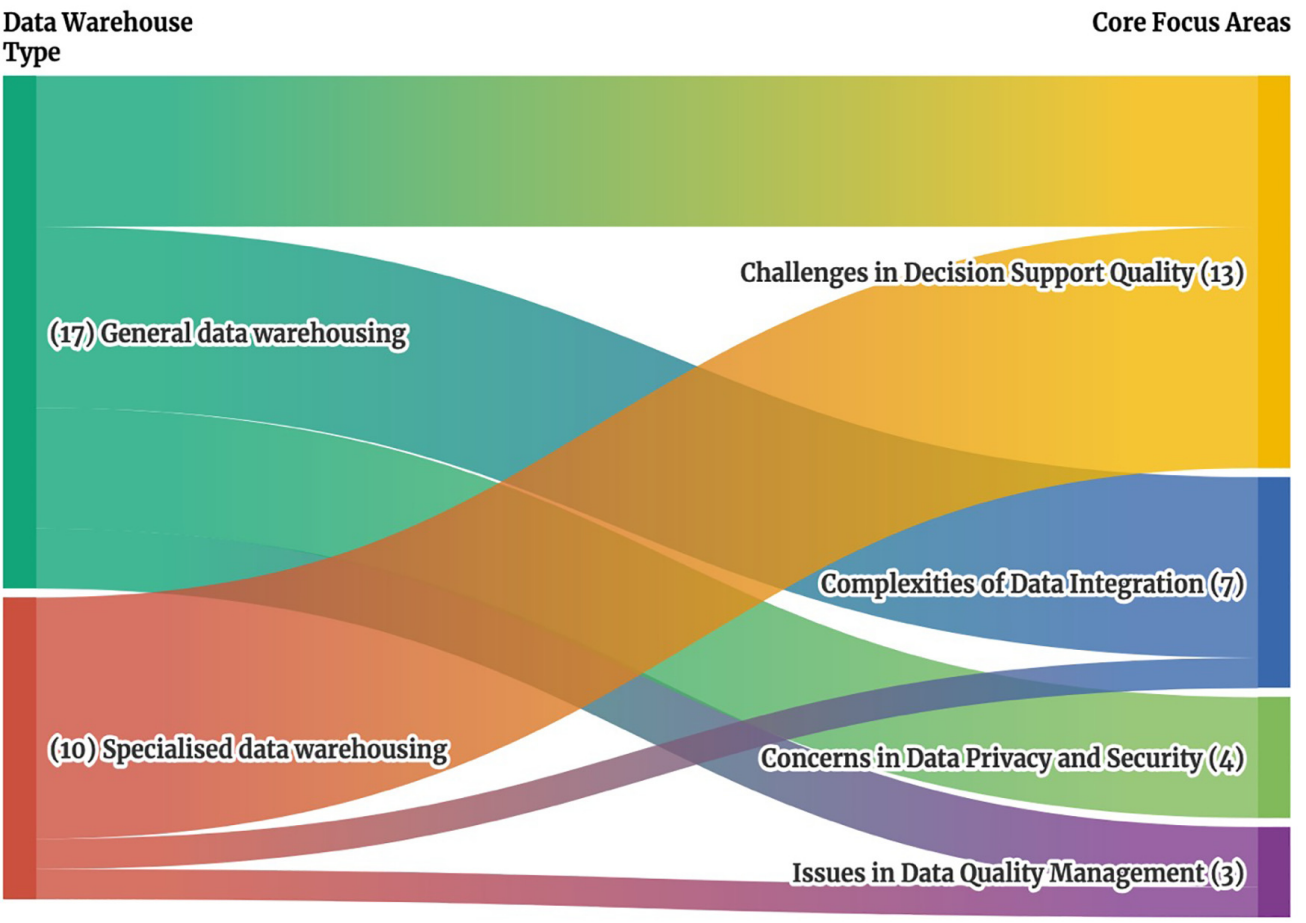
Sankey diagram of clinical data warehouse types and associated core focus areas. This Sankey diagram illustrates the relationship between clinical data warehouse types on the left, which include general specialized data warehouses, and their associated core focus areas on the right, which include challenges in decision support quality, complexities of data integration, concerns in data privacy and security and issues in data quality management. The width of each stream represents the number of studies addressing each connection, providing a visual overview of how different data warehouse types align with specific cores focus areas.

*Challenges in Decision Support Quality* were frequently cited as shown in Figure 2 appearing in 13 of 27 papers with 8 papers from Specialized data warehousing category (5, 6, 24–29) and 5 papers from General data warehousing (3, 14, 17, 19, 22). All 8 studies in the Specialized Data Warehousing category (5, 6, 24–29) highlight the specific needs of clinical data warehouses, as general-purpose warehouses lack sufficient analytical capabilities for specific diseases or unstructured data, such as medical images and text-based prescriptions. In the general data warehousing category, 4 studies focus on improving disease and patient management (14, 17, 19, 22), while one study focuses on improving data retrieval and execution times across hospital systems (3).

*Complexities of Data Integration* were cited in 7 papers (1, 8, 15, 16, 18, 21, 23). 6 studies in the General Data Warehousing category (1, 8, 15, 16, 18, 21) highlight the lack of a unified analytics system for consolidating disparate data sources, including EHR, hospital information systems (HIS), patient management systems (PMS), pharmacy information systems (PIS), and others. Only 1 study from the Specialized Data Warehousing category (23) focuses on the integration of different types of EHR data.

In *Concerns in Data Privacy and Security*, 4 papers (4, 11, 12, 20) all with a General data warehousing focus, emphasized the need for effective governance, regulatory compliance, and robust security measures, such as the US Health Insurance Portability and Accountability Act (HIPAA), and implementing security measures (20). Lastly, for Issues in *Data Quality Management*, 2 papers from General data warehousing addressed the inconsistent use and coding due to different EHR system (2) and secondary analysis use (13), while 1 paper from Specialized data warehousing focused on the accuracy of treatment data in the Cancer Research Network Virtual Data Warehouse (7).

#### 3.1.2 Data sources

**Figure 3:**
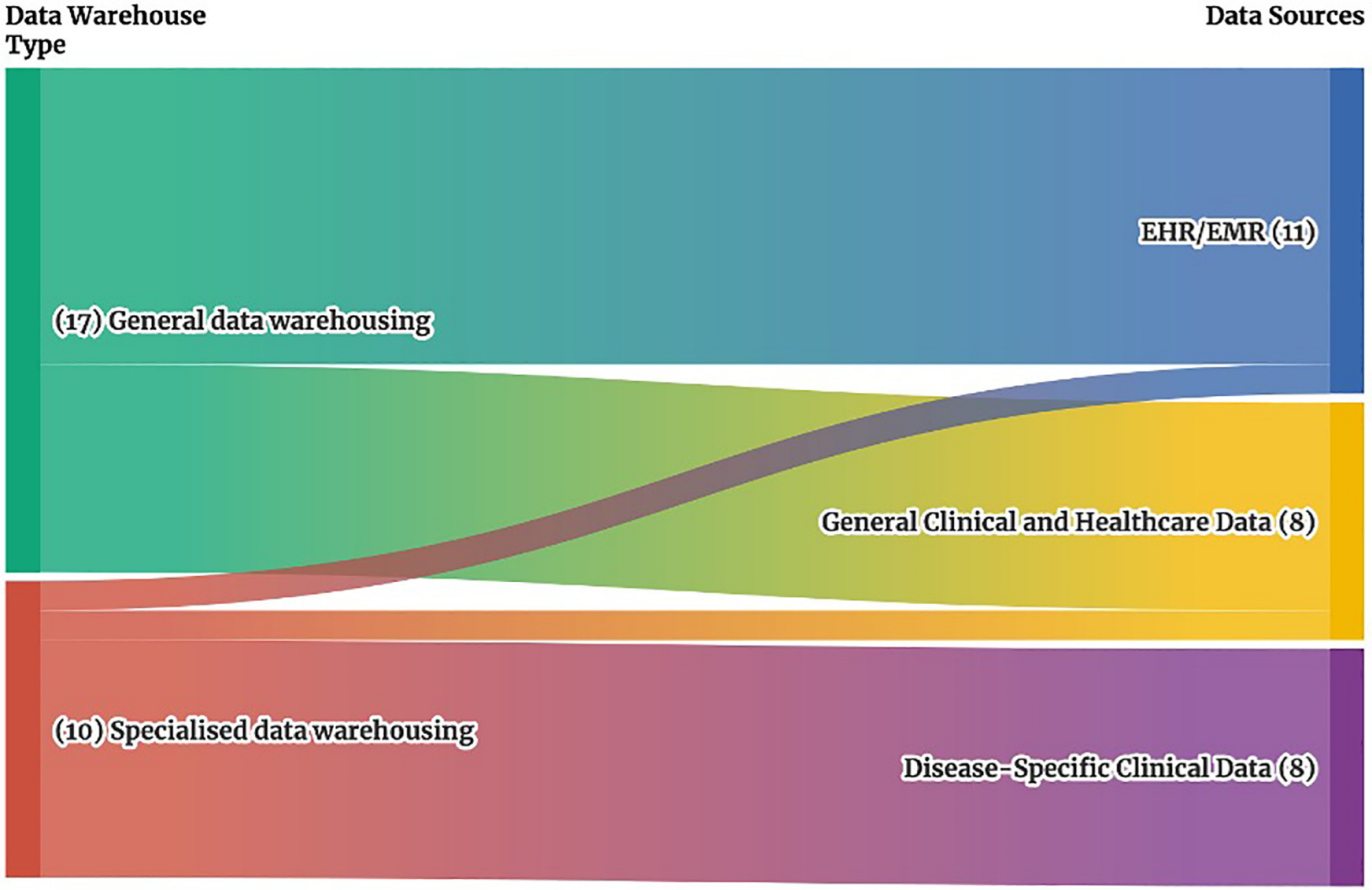
Sankey diagram of clinical data warehouse types and associated data sources. This Sankey diagram illustrates the relationship between clinical data warehouse types on the left, which include general specialized data warehouses, and their associated data sources on the right, which include EMR/EHR systems, general clinical and health data, and disease-specific clinical data. The width of each stream represents the number of studies addressing each connection, providing a visual overview of how different data warehouse types align with specific data sources.

In the General data warehousing category (Refer to Figure 3), the studies relied on *EHR/EMR* data (10 out of 17 studies) (1–3, 11–14, 17, 18, 21) and *general clinical and health data* (7 out of 17 studies) (4, 8, 15, 16, 19, 20, 22) obtained from hospital information systems (HIS), patient management systems (PMS), and pharmacy information systems (PIS), covering hospital-wide information.

In contrast, Specialized data warehousing focused primarily on *disease-specific data* (8 of 10 studies) (5–7, 23, 25, 26, 29), obtained from radiology information systems (RIS), picture archiving and communication systems (PACS), and other specialized systems focused on specific types of data or diseases. Then, one study in these specialized data warehouses analysed EHR/EMR data focusing on clinical and pathology reports (24), while another study examined general clinical and health data related to ICU processes (28).

#### 3.1.3 Analytic capabilities

As shown in Table 2, *Query analysis and OLAP* were the most frequently mentioned analytical capabilities in clinical data warehouses, cited in 11 of 27 papers, with 8 from General data warehousing (1, 3, 4, 11, 15, 17, 18, 22) and 3 from Specialized data warehousing (6, 23, 28). Among the General data warehousing, these methods were used for Medicare resource management (3, 4), health planning (11), and improving clinical outcomes (11, 17, 18, 22). In contrast, Specialized data warehousing focused on disease-specific applications, including cancer (6), obesity (23), and intensive care unit management (28). *Data mining and AI technology* was used in 5 studies (5, 16, 24, 25, 29). Only one General data warehousing study used data mining to support clinical decision-making in Bangladesh (16), while the remaining 4 specialized data warehousing studies focused on unstructured data, including image-based analysis (5, 25) and text-based data processing (24, 29). In addition, 2 Specialized data warehousing studies used *spatio-temporal analysis* for COVID-19 transmission tracking (26, 27). Finally, 9 papers did not explicitly mention the analytical methods used.

**Table 2:** Summary of the analytical capabilities perspective in the selected studies included in the scoping review.

Analytic capabilities perspectives	General data warehousing	Specialized data warehousing	Number of papers (*n* *=* *27*)
Query Analysis & OLAP	8 papers (1, 3, 4, 11, 15, 17, 18, 22)	3 papers (6, 23, 28)	11
Data Mining & AI Technology	1 paper (16)	4 papers (5, 24, 25, 29)	5
Spatio-Temporal analysis	N/A	2 papers (26, 27)	2
Not explicitly mentioned	8 papers (2, 12–14, 16, 19–21)	1 paper (7)	9

OLAP stands for Online Analytical Process, while AI stands for Artificial Intelligence.

### 3.2 Technical aspects of clinical data warehousing: data structures, and data warehouse architecture and transformation

#### 3.2.1 Data structures

Data structures are divided into aggregable and non-aggregable data, as shown in Table 3(a). The *aggregable data* is used in most (18/27) of the studies. Within 18 papers, 12 papers fall in the General data warehousing category, which use EHR/EMR and general clinical and health data for medication use (11), improving ETL process (8), enhancing medical resource allocation (1, 3, 4, 13, 14, 21), and improving clinical outcomes for patients (16–18, 22). The 6 papers from Specialized data warehousing are focused on the use of aggregable data for disease-specific analysis (6, 7, 23, 26–28). For the *non-aggregable data*, all 4 studies are from Specialized data warehousing category, which include text-based data, images and other formats (5, 24, 25, 29). Finally, 5 papers do not explicitly mention the data structures.

**Table 3:** Overall summary of (a) data structures, (b) transformation and (c) data warehouse architecture perspective in the selected studies included in the scoping review.

Perspectives	General data warehousing	Specialized data warehousing	Number of papers (*n* *=* *27*)
(a) Data structures Perspectives			
Aggregable data	12 papers (1, 3, 4, 8, 11, 13, 14, 16–18, 21, 22)	6 papers (6, 7, 23, 26–28)	18
Non-aggregable data	N/A	4 papers (5, 24, 25, 29)	4
Not explicitly mentioned	5 papers (2, 12, 15, 19, 20)	N/A	5
(b) Transformation Perspectives			
ETL taxonomy	1 papers (8)	N/A	1
Disease-specific ETL	N/A	2 papers (24, 25)	2
Process-specific ETL	N/A	1 papers (28)	1
Not explicitly mentioned	16 papers (1–4, 11–22)	7 papers (5–7, 23, 26, 27)	23
(c) Architecture Perspectives			
Star schema	7 papers (1, 3, 4, 11, 15, 16, 18)	2 papers (23, 28)	9
Specific architecture			
• Disease-specific architecture	N/A	4 papers (7, 24, 26, 27)	4
• Federated data warehouse	2 papers (17, 22)	N/A	2
• Graph data warehouse	N/A	2 papers (5, 25)	2
• Specialized warehouse for prescribing data	N/A	2 papers (24, 29)	2
Not explicitly mentioned	8 papers (2, 6, 8, 12–14, 19–21)	N/A	8

#### 3.2.2 Transformation process

There are four papers which discussed the ETL transformation process (8, 24, 25, 28), as shown in Table 3(b). Only 1 study from the General Data Warehousing category discussed the *ETL taxonomy*, which classifies ETL processes based on their frequency (8): (1) On-demand ETL is a traditional ETL process that is executed based on specific needs, with data processing occurring as needed. (2) Near real-time ETL is a faster method than on-demand ETL, with shorter processing times, and can be performed several times a day. (3) Real-time ETL, data is continuously produced and processed from data sources, allowing for immediate integration and analysis. In the Specialized data warehousing category, three studies highlighted that certain *disease analytics or processes*, such as image and text-based data, require longer ETL processing times due to their complexity (24, 25). And an ICU study found that ETL frequency positivity impacted performance analysis as frequency increased, affecting patient readmission rates, resource utilization, and timely care decisions (28).

#### 3.2.3 Data warehouse architecture

The *star schema* was the most common architecture identified in 9 studies, with 7 studies falling into the General Data Warehousing category (1, 3, 4, 11, 15, 16, 18) and 2 studies in the Specialized Data Warehousing category (23, 28). These studies were primarily designed to manage EHR/EMR and general clinical data (see Table 3(c). In addition, 2 General data warehousing studies used *federated data warehouse* approaches to integrate multiple autonomous databases (17, 22). In contrast, 4 Specialized data warehousing studies focused on *disease-specific architectures* for COVID-19 (26, 27) and cancer data (7, 24), while 2 studies introduced *graph-based warehouses* for medical imaging (25) and acute kidney injury (AKI) (5). Another 2 studies developed specialized warehouses for *prescription data* (24, 29). Finally, 8 papers did not specify their data warehouse architecture.

### 3.3 Post-implementation challenges

This section synthesizes 27 selected papers to highlight post-implementation challenges, as opposed to the core focus areas that explore broader research topics. These challenges fall into three main categories, as shown in Table 4. The remaining 5 papers didn't mention post-implementation challenges.

(1) *Automation failures* (12 papers), with 8 General data warehousing studies (1–3, 8, 14–16, 21) and 4 Specialized data warehousing studies (5, 23, 27, 28). Failures include time-consuming integration across hospitals and health systems (1, 3, 5, 14, 15, 27), inconsistent data collection requirements (8, 28), and variations in data formats and coding structures (2, 21, 23).
(2) *Data quality and privacy issues* (5 papers), with 4 General data warehousing studies (4, 12, 20, 22) and 1 Specialized data warehousing study (7). Issues included privacy policy differences by region (4), protection of sensitive patient data (12, 20), lack of validation methods for automated data (7), and challenges in assessing the quality of disease-specific data (22).
(3) *Unclear user requirements* (5 papers), noted in 3 General data warehousing studies (11, 18, 19) and 2 Specialized data warehousing studies (6, 26). Issues include unclear decision support objectives leading to information overload (11, 18, 19) and difficulties in integrating multiple subsystems to define clear objectives (6, 19, 26).

**Table 4:** Overall summary of post-implementation challenges described in the selected studies.

Post-implementation challenges	General data warehousing	Specialized data warehousing	Number of papers (*n* *=* *27*)
Automation failures	8 papers (1–3, 8, 14–16, 21)	4 papers (5, 23, 27, 28)	12
Data quality and privacy issues	4 papers (4, 12, 20, 22)	1 paper (7)	5
Unclear user requirements	3 papers (11, 18, 19)	2 papers (6, 26)	5
Not explicitly mentioned	2 papers (13, 17)	3 papers (24, 25, 29)	5

## 4 Discussion

With the growing importance of data warehousing in clinical and healthcare domains, several researchers have conducted scoping reviews of clinical data warehousing recently. These reviews have generally classified clinical data warehouses based on their focus area, application area, architecture, data model, or data domain (30–32). In comparison, this study provides a more comprehensive perspective by including an analysis of the analytical capabilities and ongoing post-implementation challenges. With the increasing demand for advanced analytics in healthcare, traditional methods such as OLAP may no longer fully meet the evolving needs of clinical data analytics. This study further classifies clinical data warehouses into two distinct evolutionary paths: General data warehousing and Specialized data warehousing, based on characteristics identified through an in-depth review of 27 selected papers. The purpose of this classification is to illustrate the different paths of general and specialized data warehouses, emphasizing the trade-off between broad hospital-wide integration and tailored, high-performance analytics for specific clinical and research applications.

### 4.1 Scalability and challenges of general data warehouses in hospitals

General data warehousing designed to enhance overall data analysis capabilities across hospitals share common characteristics. By consolidating data from multiple sources, such as EMR/EHR, lab results, and patient monitoring devices, healthcare providers can gain a more complete and nuanced understanding of each patient's health. This holistic view enables early detection of potential problems, personalised treatment plans and more effective management of chronic conditions (17).

General data warehouse implementations often rely on relational databases with a star schema architecture (1, 2). A similar study, Clinical Data Warehouse Scoping Review (31), found that the key strength of using relational database modelling is the ability to integrate a wide range of data sources from different hospital systems with the same modelling approach, providing strong data consistency, integrity, and the ability to perform complex queries, including patient records, lab results, billing information, and more, into a unified system, which is essential for comprehensive data analysis and reporting. This underscores the importance of aligning general data warehouse implementations with the World Heath Organisation's Digital Health Guidelines (33), which emphasize robust governance, standardized privacy, and interoperability to ensure the secure, consistent, and sustainable use of clinical data within complex healthcare infrastructures.

From the data analytics perspective, general data warehousing provides more comprehensive analysis than smaller, more specialised data warehouses due to its scalability. By embedding OLAP functionality, general data warehouses have been instrumental in providing powerful analytical capabilities (6). These capabilities allow users to explore various factors through multidimensional queries, including drill-down, roll-up, and slicing and dicing of data.

In comparison to other scoping reviews (30–32), this study found that general purpose data warehouses underperform their specialized counterparts in terms of analytical capabilities. This is attributed to the complexity and volume of data to be processed (1, 2), such as text and images. These limitations highlight the need for more adaptable and efficient data warehouse architectures to meet the dynamic and diverse requirements of clinical data analysis. Moreover, it was observed that the general data warehouse exhibited a lack of flexibility. In specific, star schemas, a common architectural model in general clinical data warehouses, are optimized for predefined, aggregable data and are less adaptable to unstructured clinical data such as free-text notes or medical images (30, 31). In addition, introducing new data sources often requires significant schema restructuring and reconfiguration of ETL processes, making them less responsive to evolving clinical and research needs (32). Once the scope and objective of a general data warehouse have been defined, it often lacks the flexibility to redirect its focus to different fields. The creation of specific data marts or lakes can enhance flexibility and analytical capabilities. However, this approach leads to a significant increase in development costs and a reduction in the level of automation due to the need to establish new ETL processes. These post-implementation challenges underscore the importance of applying structured implementation frameworks to clinical data warehousing. A recent study (34) has shown that data warehouse systems require adaptive system design, iterative implementation, and stakeholder engagement to evolve with changing clinical priorities, support diverse data types, and be sustainable in dynamic healthcare settings.

### 4.2 Specialized data warehouses and their challenges

As specialised data warehouses have a narrow and clear scope defined by their subject matter, developers have more freedom to choose suitable and appropriate architectures that focus on specific areas. A recent scoping review also indicated that the specific requirements for the clinical data warehouse are increasing, driven by the complexity of the data (30). For instance, the researchers proposed a NoSQL-based data warehouse specifically designed for graph analysis because of its ability to explore data and its agility in representing clinical facts related to acute kidney injury (5). Another study proposed a new ETL structure embedded with a perceptual layer to detect the similarity of medical images (25). Due to the clear design boundaries, the ETL process of the specialised data warehouse also appeared to be more effective. Meanwhile, the topics and data structure for specialised data warehousing vary considerably. Of the 27 selected papers, 8 proposed specialised data warehousing focusing on areas such as obesity (23), cancer (6, 7), acute kidney injury (5), medical image-based diagnosis (25) and COVID-19 (26, 27).

Based on the classification, specialised data warehousing appears to demonstrate superior analytical performance compared to general data warehouses. These models not only support OLAP but also incorporate advanced data mining and AI tools tailored to specific characteristics of diseases or task areas. For instance, the COVID-19 data warehouses proposed in 2020 and 2022 (14, 15) were enhanced with spatial clustering analysis to detect patterns of virus transmission. In addition, another study applied an NLP model to analyse text-based data, such as prescriptions and clinical notes, in a clinical setting (29). This capability addresses a significant limitation of general data warehouses, which often lack adequate analytical tools for unstructured data. These advances highlight the potential for specialised data warehouses to provide more targeted and effective analytical capabilities, using advanced AI and data mining techniques to address the unique challenges of specific clinical applications.

Furthermore, AI based studies have reported technical metrics such as RMSE, precision, and recall validating AI models within specialized data warehouses (5, 16, 24, 25, 29). However, these evaluations have typically been limited to single-site or context-specific datasets. As a result, the generalizability and clinical applicability of these models remains uncertain (34). What works well in one institutional setting may not translate effectively to others due to differences in data quality, patient populations, or clinical practices. In addition, Ethical issues such as potential bias in training data and transparency of model selection are also rarely discussed (34). This underscores the need for standardized frameworks that ensure rigorous validation of AI models, clinical readiness through external testing. The EU AI Act (35) is an example of a regulatory initiative that mandates transparency and accountability in AI systems. The US ONC Interoperability Framework (36) also promotes data standardization and secure exchange. Together, these initiatives highlight the importance of aligning AI-enabled clinical data warehouses with evolving policies to ensure the ethical and trustworthy adoption of scalable healthcare.

Despite potential analytic advantages, specialised data warehousing often involve a narrow focus, which can limit scalability and make it difficult to determine which patients or patient groups should be included. Given the complexity of the clinical environment, the diagnosis and treatment of disease is a multifaceted process influenced by many factors. Integrating multiple perspectives and data sources can significantly improve the accuracy and success of diagnosis and treatment. However, specialised data warehousing is not easily able to accommodate additional data sources or new types of data outside their original scope, as specific architectures and customised ETL processes that make specialised warehouses effective for specific tasks may not be compatible with more general systems, leading to data silos and interoperability issues. To address the scalability limitations of specialized data warehouses, recent studies (37–39) have proposed flexible architectures as potential evolutionary paths. Two studies (37, 38) have proposed hybrid models that integrate centralized data lakes with disease-specific marts to increase adaptability without sacrificing analytical depth. A study (39) in 2023 proposes federated learning to enable collaborative analysis across institutions without centralizing sensitive data, supporting both scalability and privacy.

Lastly, in addition to providing a comparative analysis of general and specialized data warehouses, this review highlights critical structural and governance challenges common to both types. Vendor lock-in can limit interoperability and increase long-term maintenance costs by tying a hospital or clinical department to a specific technology vendor (1). Meanwhile, governance silos can prevent data sharing due to fragmented policies (40, 41), especially for large clinical data warehouses that span more than one hospital. Another barrier is the regulatory discrepancy between data privacy laws (4), as different countries have different regulations, such as HIPAA in the United States (20) and GDPR in Europe (42), which complicates cross-border implementation. These issues impact scalability, compliance, and data utility. Addressing them requires coordinated policies, unified data governance models, and interoperable standards to support the secure and ethical use of data warehouses across healthcare systems.

### 4.3 Strengths and limitations

This scoping review provides a comprehensive analysis of clinical data warehousing from the perspective of both general and specialized data warehouses, offering a structured comparison of their core focus areas, data sources, analytic capabilities, and data warehouse transformation and architecture, as well as post-implementation changes. The study employs a variety of analytical methods, including Sankey diagrams, tabular summaries, and narrative synthesis, to effectively present key findings. In addition, by examining core functional aspects, technical structures, and post-implementation changes, this review highlights critical gaps and future directions in clinical data warehousing.

There are several limitations to this scoping review. Firstly, the exclusion of non-English studies may introduce a language bias. This could lead to the omission of valuable research conducted in other languages. Secondly, the reliance on pre-defined keywords for the literature search may have resulted in the exclusion of relevant studies that were not captured by the search terms. This may have limited the comprehensiveness of the review. Despite these limitations, this scoping review provides valuable insights into using Data Warehouses in clinical practice. Thirdly, four major databases-PubMed, CINAHL, Scopus, and IEEE Xplore-were selected to ensure broad interdisciplinary coverage, but this approach may not have included all relevant literature. Future work will seek to include additional databases, such as the ACM Digital Library and Web of Science, to further improve the comprehensiveness of the search strategy. Lastly, this review did not explicitly evaluate how clinicians adapted, used, or integrated data warehouses into daily clinical practice. Future research is needed to explore the engagement of end users and the practical application of these systems in the real world of clinical practice.

## 5 Conclusion

This scoping review study provides a comprehensive analysis of data warehouse development in the clinical and healthcare domains. Both general and specialised data warehousing enhance data analysis capabilities for clinical users.

Overall, the data warehouse is a valuable analytical tool for the clinical sector, providing comprehensive data integration and decision support. However, its characteristics and functionality vary depending on the type of data warehouse. General data warehousing, commonly used in clinical environments, integrate disparate data sources to provide a comprehensive view of patient care and resource management. However, they often lack flexibility and struggle with complex ETL processes and handling unstructured data. Specialised data warehousing, on the other hand, offer greater flexibility and advanced analytical capabilities such as AI and data mining, but face scalability and integration challenges. Continued innovation in design and architecture is needed to address these limitations and maximise their effectiveness in clinical settings.

To support future development, hybrid data warehouse architectures, such as combining central data lakes with domain-specific data marts and federation learning, offer potentially effective approaches to achieve the balance between scale and specialization. In addition, ensuring consistent validation of AI models and promoting secure and interoperable data sharing can be achieved through the adoption of standardized frameworks such as the EU Artificial Intelligence Act and the US ONC Interoperability Framework. These practices are essential to enable trusted AI integration, cross institutional collaboration, and the long-term sustainability of clinical data warehousing systems.

## References

[1] Priou S Lamé G Jankovic M Kempf E. “In conferences, everyone goes ‘health data is the future’”: an interview study on challenges in re-using EHR data for research in clinical data warehouses. AMIA Annu Symp Proc. (2024) 2023:579–88. 38222365 PMC10785853

[2] Henley-Smith S Boyle D Gray K. Improving a secondary use health data warehouse: proposing a multi-level data quality framework. eGEMs. (2019) 7(1):38. 10.5334/egems.298 31531384 PMC6676919

[3] Wade TD Hum RC Murphy JR. A dimensional bus model for integrating clinical and research data. J Am Med Inform Assoc. (2014) 18(Suppl 1):102. 10.1136/amiajnl-2011-000339 PMC3241170 21856687

[4] Sebaa A Nouicer A Tari A Tarik R Abdellah O. Decision support system for health care resources allocation. Electron Physician. (2017) 9(6):4661. 10.19082/4661 28848645 PMC5557150

[5] Baghal A Al-Shukri S Kumari A. Agile natural language processing model for pathology knowledge extraction and integration with clinical enterprise data warehouse. 2019 Sixth International Conference on Social Networks Analysis, Management and Security (SNAMS); IEEE (2019). p. 419–22

[6] Atay CE Garani G. Building a lung and ovarian cancer data warehouse. Healthc Inform Res. (2020) 26(4):303. 10.4258/hir.2020.26.4.303 33190464 PMC7674817

[7] Ritzwoller DP Carroll N Delate T O’Keeffe-Rossetti M Fishman PA Loggers ET Validation of electronic data on chemotherapy and hormone therapy use in HMOs. Med Care. (2014) 51(10):e67–73. 10.1097/mlr.0b013e31824def85 PMC3406224 22531648

[8] De Assis Vilela F Times VC de Campos Bernardi AC de Paula Freitas A Ciferri RR. A non-intrusive and reactive architecture to support real-time ETL processes in data warehousing environments. Heliyon. (2023) 9(5):e15728. 10.1016/j.heliyon.2023.e15728 37215774 PMC10196447

[9] Tricco AC Lillie E Zarin W O'Brien KK Colquhoun H Levac D PRISMA Extension for scoping reviews (PRISMA-ScR): checklist and explanation. Ann Intern Med. (2018) 169(7):467–73. 10.7326/M18-0850 30178033

[10] PyMed. PyMed: Python Package for Medical Records. Beaverton, OR: Python Software Foundation (n.d.). Available at: https://pypi.org/project/pymed/ (Accessed July 12, 2024)

[11] Freund J Meiman J Kraus C. Using electronic medical record data to characterize the level of medication use by age-groups in a network of primary care clinics. J Prim Care Community Health. (2014) 4(4):286–93. 10.1177/2150131913495243 24327665

[12] Puppala M He T Yu X Chen S Ogunti R Wong STC. Data security and privacy management in healthcare applications and clinical data warehouse environment. 2016 IEEE-EMBS International Conference on Biomedical and Health Informatics (BHI); IEEE (2016). 10.1109/bhi.2016.7455821

[13] Nobles AL Vilankar K Wu H Barnes LE. Evaluation of data quality of multisite electronic health record data for secondary analysis. 2015 IEEE International Conference on big Data (big Data); IEEE (2015). p. 2612–20

[14] Neamah AF. Flexible data warehouse: towards building an integrated electronic health record architecture. 2020 International Conference on Smart Electronics and Communication (ICOSEC); IEEE (2020). p. 1038–42

[15] Krause DD. Data lakes and data visualization: an innovative approach to address the challenges of access to health care in Mississippi. Online J Public Health Inform. (2015) 7(3):e61708. 10.5210/ojphi.v7i3.6047 PMC4731224 26834938

[16] Khan SI Hoque ASML. Towards development of health data warehouse: Bangladesh perspective. 2015 International Conference on Electrical Engineering and Information Communication Technology (ICEEICT); IEEE (2015). p. 1–6

[17] McGlothlin JP Vedire S Crawford E Pappas J Bruneau B Obregon L. Improving patient care through analytics. 2016 4th International Symposium on Computational and Business Intelligence (ISCBI); IEEE (2016). p. 94–100

[18] Abouzahra M Sartipi K Armstrong D Tan J. Integrating data from EHRs to enhance clinical decision making: the inflammatory bowel disease case. 2014 IEEE 27th International Symposium on Computer-Based Medical Systems; IEEE (2014). p. 531–2

[19] Shin SY Kim WS Lee JH. Characteristics desired in clinical data warehouse for biomedical research. Healthc Inform Res. (2014) 20(2):109. 10.4258/hir.2014.20.2.109 24872909 PMC4030054

[20] Faridoon A Kechadi MT. Data behind the walls—an advanced architecture for data privacy management. 2022 International Conference on Computational Science and Computational Intelligence (CSCI); IEEE (2022). p. 922–8

[21] Ren S Wang T Lu X. Dimensional modeling of medical data warehouse based on ontology. 2018 IEEE 3rd International Conference on Big Data Analysis (ICBDA); IEEE (2018). p. 144–9

[22] Karami M Rahimi A Shahmirzadi AH. Clinical data warehouse: an effective tool to create intelligence in disease management. Health Care Manag (Frederick). (2017) 36(4):380–4. 10.1097/HCM.0000000000000113 28938242

[23] Wood GC Chu X Manney C Strodel W Petrick A Gabrielsen J An electronic health record-enabled obesity database. BMC Med Inform Decis Mak. (2016) 12(1):45. 10.1186/1472-6947-12-45 PMC3508953 22640398

[24] Baghal A. Leveraging graph models to design acute kidney injury disease research data warehouse. 2019 Sixth International Conference on Social Networks Analysis, Management and Security (SNAMS); IEEE (2019). p. 413–8

[25] Teixeira JW Annibal LP Felipe JC Ciferri RR de Aguiar Ciferri CD. A similarity-based data warehousing environment for medical images. Comput Biol Med. (2015) 66:190–208. 10.1016/j.compbiomed.2015.08.019 26414378

[26] Artemova S Caporossi A Cancé C Madiot PE Nemoz B Larrat S COVID-19 geographical maps and clinical data warehouse PREDIMED. In: Otero P, Scott P, Martin SZ, Huesing E, editors. MEDINFO 2021: One World, One Health—Global Partnership for Digital Innovation. Amsterdam: IOS Press (2022). p. 1046–7. 10.3233/SHTI220260 35673198

[27] Agapito G Zucco C Cannataro M. COVID-warehouse: a data warehouse of Italian COVID-19, pollution, and climate data. Int J Environ Res Public Health. (2020) 17(15):5596. 10.3390/ijerph17155596 32756428 PMC7432400

[28] Scheer J Nagel T Ganslandt T. A visual approach for analyzing readmissions in intensive care medicine. In: Proceedings of the 2020 Workshop on Visual Analytics in Healthcare (VAHC); 2020 Oct 25; Chicago, IL, United States. Piscataway, NJ: IEEE (2020). p. 24–5. 10.1109/VAHC53729.2020.00010

[29] Grammatico-Guillon L Shea K Jafarzadeh SR Camelo I Maakaroun-Vermesse Z Figueira M Antibiotic prescribing in outpatient children: a cohort from a clinical data warehouse. Clin Pediatr (Phila). (2019) 58(6):681–90. 10.1177/0009922819834278 30884973

[30] Wang Z Craven C Syed M Greer M Seker E Syed S Clinical data warehousing: a scoping review. J Soc Clin Data Manag. (2024) 4(1):1–19. 10.47912/jscdm.320 PMC12834563 41602775

[31] Zhang H Lyu T Yin P Bost S He X Guo Y A scoping review of semantic integration of health data and information. Int J Med Inform. (2022) 165:104834. 10.1016/j.ijmedinf.2022.104834 35863206

[32] Shau WY Santoso H Jip V Setia S. Integrated real-world data warehouses across 7 evolving Asian health care systems: scoping review. J Med Internet Res. (2024) 26:e56686. 10.2196/56686 38749399 PMC11200047

[33] World Health Organization. Digital Health. Geneva: World Health Organization (2025). Available at: https://www.who.int/health-topics/digital-health#tab=tab_1 (Accessed April 21, 2025)

[34] Nilsen P Svedberg P Neher M Nair M Larsson I Petersson L A framework to guide implementation of AI in health care: protocol for a cocreation research project. JMIR Res Protoc. (2023) 12(1):e50216. 10.2196/50216 37938896 PMC10666006

[35] European Commission. EU Artificial Intelligence Act. Brussels: European Commission (2024). Available at: https://artificialintelligenceact.eu/ (Accessed April 21, 2025)

[36] U.S. Office of the National Coordinator for Health Information Technology (ONC). Interoperability. Washington, DC: U.S. Department of Health and Human Services (2025). Available at: https://www.healthit.gov/topic/interoperability (Accessed April 21, 2025)

[37] Gentner T Neitzel T Schulze J Gerschner F Theissler A. Data lakes in healthcare: applications and benefits from the perspective of data sources and players. Procedia Comput Sci. (2023) 225:1302–11. 10.1016/j.procs.2023.10.118

[38] Maini E Venkateswarlu B Gupta A. Data lake-an optimum solution for storage andanalytics of big data in cardiovascular disease prediction system. Int J Comput Eng Manag (IJCEM). (2018) 21:33–9.

[39] Oh W Nadkarni GN. Federated learning in health care using structured medical data. Adv Kidnzy Dis Health. (2023) 30(1):4–16. 10.1053/j.akdh.2022.11.007 PMC10208416 36723280

[40] Holmes JH Elliott TE Brown JS Raebel MA Davidson A Nelson AF Clinical research data warehouse governance for distributed research networks in the USA: a systematic review of the literature. J Am Med Inform Assoc. (2014) 21(4):730–6. 10.1136/amiajnl-2013-002370 24682495 PMC4078282

[41] Knosp BM Craven CK Dorr DA Bernstam EV Campion TR Jr. Understanding enterprise data warehouses to support clinical and translational research: enterprise information technology relationships, data governance, workforce, and cloud computing. J Am Med Inform Assoc. (2022) 29(4):671–6. 10.1093/jamia/ocab256 35289370 PMC8922193

[42] Mulder T Tudorica M. Privacy policies, cross-border health data and the GDPR. Inf Commun Technol L. (2019) 28(3):261–74. 10.1080/13600834.2019.1644068

