# Supplementary material for: The development and use of data warehousing in clinical settings: a scoping review
Source: Front Digit Health. 2025 Jun 5;7:1599514. doi: 10.3389/fdgth.2025.1599514 (PMC12176853; doi:10.3389/fdgth.2025.1599514)
Supplement: Supplementary file 1 [file Datasheet1.pdf]

Supplementary Material

SECTION	ITEM	PRISMA-ScR CHECKLIST ITEM	Reported in Section
**TITLE**			
Title	1	Identify the report as a scoping review.	Title page
**ABSTRACT**			
Structured summary	2	Provide a structured summary that includes (as applicable): background, objectives, methods, results, and conclusions that relate to the review questions and objectives.	Abstract
**INTRODUCTION**			
Rationale	3	Describe the rationale for the review in the context of what is already known. Explain why the review questions/objectives lend themselves to a scoping review approach.	Introduction
Objectives	4	Provide an explicit statement of the questions and objectives being addressed with reference to their key elements (e.g., population or participants, concepts, and context) or other relevant key elements used to conceptualize the review questions and/or objectives.	Introduction
**METHODS**			
Protocol and registration	5	Indicate whether a review protocol exists; state if and where it can be accessed (e.g., a Web address); and if available, provide registration information, including the registration number.	Method
Eligibility criteria	6	Specify characteristics of the sources of evidence used as eligibility criteria (e.g., years considered, language, and publication status), and provide a rationale.	Method - 2.2 Inclusion and Exclusion criteria
Information sources	7	Describe all information sources in the search (e.g., databases with dates of coverage and contact with authors to identify additional sources), as well as the date the most recent search was executed.	Method - 2.1 Information Sources and Search Strategy
Search	8	Present the full electronic search strategy for at least 1 database, including any limits used, such that it could be repeated.	Method - 2.1 Information Sources and Search Strategy<br>&<br><br>Supplementary table 2
Selection of sources of evidence	9	State the process for selecting sources of evidence (i.e., screening and eligibility) included in the scoping revie	Method -2.3 Study Selection and Data Extraction

Data charting process	10	Describe the methods of charting data from the included sources of evidence (e.g., calibrated forms or forms that have been tested by the team before their use, and whether data charting was done independently or in duplicate) and any processes for obtaining and confirming data from investigators.	Method - 2.3 Study Selection and Data Extraction<br><br>&<br><br>Supplementary table 2 and 3
Data items	11	List and define all variables for which data were sought and any assumptions and simplifications made.	Method - 2.3 Study Selection and Data Extraction
Critical appraisal of individual sources of evidence	12	If done, provide a rationale for conducting a critical appraisal of included sources of evidence; describe the methods used and how this information was used in any data synthesis (if appropriate).	N/A
Synthesis of results	13	Describe the methods of handling and summarizing the data that were charted.	Method - 2.4 Data Synthesis
**RESULTS**			
Selection of sources of evidence	14	Give numbers of sources of evidence screened, assessed for eligibility, and included in the review, with reasons for exclusions at each stage, ideally using a flow diagram.	Results
Characteristics of sources of evidence	15	For each source of evidence, present characteristics for which data were charted and provide the citations.	Results
Critical appraisal within sources of evidence	16	If done, present data on critical appraisal of included sources of evidence (see item 12).	N/A
Results of individual sources of evidence	17	For each included source of evidence, present the relevant data that were charted that relate to the review questions and objectives.	Results
Synthesis of results	18	Summarize and/or present the charting results as they relate to the review questions and objectives.	Results
**DISCUSSION**			
Summary of evidence	19	Summarize the main results (including an overview of concepts, themes, and types of evidence available), link to the review questions and objectives, and consider the relevance to key groups.	Discussion
Limitations	20	Discuss the limitations of the scoping review process.	Discussion
Conclusions	21	Provide a general interpretation of the results with respect to the review questions and objectives, as well as potential implications and/or next steps.	Discussion & Conclusion
**FUNDING**			

Funding	22	Describe sources of funding for the included sources of evidence, as well as sources of funding for the scoping review. Describe the role of the funders of the scoping review.	N/A

Supplementary Table 1: PRISMA-SCR checklist. This scoping review study adheres to the established Preferred Reporting Items for Systematic Reviews and Meta-Analyses (PRISMA) guidelines.

Database	Query	Result
PubMed	("english"[Language] AND ("data warehousing"[MeSH Terms] OR ("data"[All Fields] AND "warehousing"[All Fields]) OR "data warehousing"[All Fields] OR ("data"[All Fields] AND "warehouse"[All Fields]) OR "data warehouse"[All Fields] OR ("data warehousing"[MeSH Terms] OR ("data"[All Fields] AND "warehousing"[All Fields]) OR "data warehousing"[All Fields])) AND ("electronic health records"[MeSH Terms] OR ("electronic"[All Fields] AND "health"[All Fields] AND "records"[All Fields]) OR "electronic health records"[All Fields] OR ("electronic"[All Fields] AND "medical"[All Fields] AND "record"[All Fields]) OR "electronic medical record"[All Fields] OR ("empir musicol rev"[Journal] OR "emr"[All Fields]) OR ("ethics hum res"[Journal] OR "environ hist rev"[Journal] OR "ehr"[All Fields]) OR ("electronic health records"[MeSH Terms] OR ("electronic"[All Fields] AND "health"[All Fields] AND "records"[All Fields]) OR "electronic health records"[All Fields] OR ("electronic"[All Fields] AND "health"[All Fields] AND "record"[All Fields]) OR "electronic health record"[All Fields]) OR (("delivery of health care"[MeSH Terms] OR ("delivery"[All Fields] AND "health"[All Fields] AND "care"[All Fields]) OR "delivery of health care"[All Fields] OR "healthcare"[All Fields] OR "healthcare s"[All Fields] OR "healthcares"[All Fields]) AND ("record s"[All Fields] OR "recordability"[All Fields] OR "recordable"[All Fields] OR "recordables"[All Fields] OR "recorded"[All Fields] OR "recorder"[All Fields] OR "recorders"[All Fields] OR "recording"[All Fields] OR "recordings"[All Fields] OR "records"[MeSH Terms] OR "records"[All Fields] OR "record"[All Fields])) OR (("health"[MeSH Terms] OR "health"[All Fields] OR "health s"[All Fields] OR "healthful"[All Fields] OR "healthfulness"[All Fields] OR "healths"[All Fields]) AND ("record s"[All Fields] OR "recordability"[All Fields] OR "recordable"[All Fields] OR "recordables"[All Fields] OR "recorded"[All Fields] OR "recorder"[All Fields] OR "recorders"[All Fields] OR "recording"[All Fields] OR "recordings"[All Fields] OR "records"[MeSH Terms] OR "records"[All Fields] OR "record"[All Fields])) OR ("medical records"[MeSH Terms] OR ("medical"[All Fields] AND "records"[All Fields]) OR "medical records"[All Fields] OR ("medical"[All Fields] AND "record"[All Fields]) OR "medical record"[All Fields]) OR (("ambulatory care facilities"[MeSH Terms] OR ("ambulatory"[All Fields] AND "care"[All Fields] AND "facilities"[All Fields]) OR "ambulatory care facilities"[All Fields] OR "clinic"[All Fields] OR "clinic s"[All Fields] OR "clinical"[All Fields] OR "clinically"[All Fields] OR "clinicals"[All Fields] OR "clinics"[All Fields]) AND ("record s"[All Fields] OR "recordability"[All Fields] OR "recordable"[All Fields] OR "recordables"[All Fields] OR "recorded"[All Fields] OR "recorder"[All Fields] OR "recorders"[All Fields] OR "recording"[All Fields] OR "recordings"[All Fields] OR "records"[MeSH Terms] OR "records"[All Fields] OR "record"[All Fields]))) AND 2014/01/01:2024/12/31[Date - Publication]) AND (2014:2024[pdat])	1017 papers
CINAHL	("data warehouse" OR "data warehosing") AND ("electronic medical record" OR "EMR" OR "EHR" OR "electronic health record" OR "health record" OR "healthcare record" OR "medical record" OR "clinical data") Limiters - Full Text; Publication Date: 20140101-20241231 Narrow by Language: - english Search modes - Proximity	32 papers
Scopus	(ALL ("data warehouse") OR ALL ("data warehousing")) AND (ALL ("electronic medical record") OR ALL ("EMR") OR ALL ("EHR") OR ALL ("electronic health record") OR ALL ("health record") OR ALL ("healthcare record") OR ALL ("medical record") OR ALL ("clinical record")) AND (PUBYEAR > 2013) AND (PUBYEAR < 2025) AND (LIMIT-TO (LANGUAGE , "English"))	4185 papers
IEEE Xplore	(((("Full Text Only":data warehous*) AND (("Full Text Only": "electronic medical record") OR ("Full Text Only": "EMR") OR ("Full Text Only": "EHR") OR ("Full Text Only": "electronic health record") OR ("Full Text Only": "health record") OR ("Full Text Only":	812 papers

	"healthcare record") OR ("Full Text Only": "medical record") OR ("Full Text Only": "clinical record"))))) Filters Applied: Conferences Journals<br><br>2014 – 2024	

Supplementary Table 2: Search strategy among four databases (PubMed, CINAHL, Scopus and IEEE-Xplore). PubMed and CINAHL specialise in health and clinical fields, while IEEE-Xplore focuses on technology and engineering research. Scopus, a general multidisciplinary database, was used to capture different perspectives on data warehousing. The search strategy categorised keywords into two domains: data warehouse technology and clinical. Papers that overlapped both domains were included.

**Attributes**	**Definition**
**General study characteries**	
Paper title	Title of paper
Authors	List of authors of the study
Publication year	Year of publication of the study
Author/publication keywords	The keywords chosen by the authors or the publication to represent the main themes of their research.
Abstract	The abstract of paper
**Data warehouses domain**	
Type of data warehouse identified	Identify the type of clinical data warehouse based on the characteristics of the implementation intention, data source and structure involved, architecture, analytical capabilities, etc., including two types: general data warehouse and specialised data warehouse.
Data source	Explore the sources of data used in the data warehouse, such as Electronic Medical Records (EMR) and Electronic Health Records (EHR), etc.
Data structure	Explore the different structure of data used in the clinical data warehouse, including relational tables, star schemas, and hierarchical structures.
Extract, Transform, Load (ETL) type	Identify the type of ETL implemented in the clinical data warehouse, including on-demand ETL, near-real-time ETL and real-time ETL, and how it affects the performance of the data warehouse.
Architecture of data warehouse	Explore the architecture of the data warehouse in the clinical environment and how it affects the performance of the clinical data warehouse, including star schema or specific architectures for particular diseases.
Analytic capabilities	Explore the analytical capabilities of the data warehouse in a clinical setting, including the analytical technology used and how it improves data analysis capabilities, including OLAP (Online Analytical Processing) modules, artificial intelligence (AI) technology.
Post-implementation Challenges	Explore the ongoing limitations of data warehousing in clinical settings, including unclear user requirements, data quality and privacy issues, and automation failures.
**Clinical domains**	
Type of diseases involved	Identify the diseases that clinical data warehouses have focused on and aimed to analyse, whether targeting specific diseases or broader disease management.

Number of Hospitals involved	Identify the number of hospitals involved in the data warehouse, including single hospital implementations, multi-hospital setups (more than 5 hospitals/clinics) and national-level systems.
Core focus area	Explore the intentions behind the implementation of data warehouses in clinical settings, including addressing decision support challenges, data integration issues, privacy concerns and data quality issues.

Supplementary Table 3: The data extraction form used to record methodological and outcome variables was collected from each study by two reviewers to ensure consistency and accuracy of data collection.

Reference

1. Priou S, Lamé G, Jankovic M, Kempf E. "In conferences, everyone goes 'health data is the future'": an interview study on challenges in re-using EHR data for research in Clinical Data Warehouses. AMIA Annu Symp Proc. 2023;2023:579.
2. Henley-Smith S, Boyle D, Gray K. Improving a secondary use health data warehouse: proposing a multi-level data quality framework. eGEMs. 2019;7(1).
3. Wade TD, Hum RC, Murphy JR. A Dimensional Bus model for integrating clinical and research data. J Am Med Inform Assoc. 2014;18(Suppl 1)i102. doi:10.1136/amiajnl-2011-000339.
4. Sebaa A, Nouicer A, Tari A, Tarik R, Abdellah O. Decision support system for health care resources allocation. Electron Physician. 2017;9(6):4661.
5. Freund J, Meiman J, Kraus C. Using Electronic Medical Record Data to Characterize the Level of Medication Use by Age-Groups in a Network of Primary Care Clinics. J Prim Care Community Health. 2014;4(4):286-293. doi:10.1177/2150131913495243.
6. Puppala M, He T, Yu X, Chen S, Ogunti R, Wong STC. Data security and privacy management in healthcare applications and clinical data warehouse environment. In: 2016 IEEE-EMBS International Conference on Biomedical and Health Informatics (BHI). IEEE; 2016. doi:10.1109/bhi.2016.7455821.
7. Nobles AL, Vilankar K, Wu H, Barnes LE. Evaluation of data quality of multisite electronic health record data for secondary analysis. In: 2015 IEEE international conference on big data (big data). IEEE; 2015. p. 2612-2620.
8. Neamah AF. Flexible data warehouse: towards building an integrated electronic health record architecture. In: 2020 International Conference on Smart Electronics and Communication (ICOSEC). IEEE; 2020. p. 1038-1042.
9. Krause DD. Data lakes and data visualization: an innovative approach to address the challenges of access to health care in Mississippi. Online J Public Health Inform. 2015;7(3).
10. Khan SI, Hoque ASML. Towards development of health data warehouse: Bangladesh perspective. In: 2015 International Conference on Electrical Engineering and Information Communication Technology (ICEEICT). IEEE; 2015. p. 1-6.
11. McGlothlin JP, Vedire S, Crawford E, Pappas J, Bruneau B, Obregon L. Improving patient care through analytics. In: 2016 4th International Symposium on Computational and Business Intelligence (ISCBI). IEEE; 2016. p. 94-100.

12. Wood GC, Chu X, Manney C, Strodel W, Petrick A, Gabrielsen J, et al. An electronic health record-enabled obesity database. BMC Med Inform Decis Mak. 2016;12(1). doi:10.1186/1472-6947-12-45.
13. Abouzahra M, Sartipi K, Armstrong D, Tan J. Integrating data from EHRs to enhance clinical decision making: the inflammatory bowel disease case. In: 2014 IEEE 27th International Symposium on Computer-Based Medical Systems. IEEE; 2014. p. 531-2.
14. Baghal A, Al-Shukri S, Kumari A. Agile natural language processing model for pathology knowledge extraction and integration with clinical enterprise data warehouse. In: 2019 Sixth International Conference on Social Networks Analysis, Management and Security (SNAMS). IEEE; 2019. p. 419-22.
15. Teixeira JW, Annibal LP, Felipe JC, Ciferri RR, de Aguiar Ciferri CD. A similarity-based data warehousing environment for medical images. Comput Biol Med. 2015;66:190-208.
16. Shin SY, Kim WS, Lee JH. Characteristics desired in clinical data warehouse for biomedical research. Healthc Inform Res. 2014;20(2):109.
17. Baghal A. Leveraging Graph Models to Design Acute Kidney Injury Disease Research Data Warehouse. In: 2019 Sixth International Conference on Social Networks Analysis, Management and Security (SNAMS). IEEE; 2019. p. 413-418.
18. Artemova S, Caporossi A, Cancé C, Madiot PE, Nemoz B, Larrat S, et al. COVID-19 geographical maps and clinical data warehouse PREDIMED. In: MEDINFO 2021: One World, One Health – Global Partnership for Digital Innovation. IOS Press; 2022. p. 1046-7.
19. Agapito G, Zucco C, Cannataro M. COVID-warehouse: A data warehouse of Italian COVID-19, pollution, and climate data. Int J Environ Res Public Health. 2020;17(15):5596.
20. Atay CE, Garani G. Building a lung and ovarian cancer data warehouse. Healthc Inform Res. 2020;26(4):303.
21. Ritzwoller DP, Carroll N, Delate T, O'Keeffe-Rossetti M, Fishman PA, Loggers ET, et al. Validation of electronic data on chemotherapy and hormone therapy use in HMOs. Med Care. 2014;51(10):e67-73. doi:10.1097/mlr.0b013e31824def85.
22. Scheer J, Nagel T, Ganslandt T. A visual approach for analyzing readmissions in intensive care medicine. In: 2020 Workshop on Visual Analytics in Healthcare (VAHC). IEEE; 2020. p. 24-5.
23. Faridoon A, Kechadi MT. Data Behind the Walls – An Advanced Architecture for Data Privacy Management. In: 2022 International Conference on Computational Science and Computational Intelligence (CSCI). IEEE; 2022. p. 922-8.
24. De Assis Vilela F, Times VC, de Campos Bernardi AC, de Paula Freitas A, Ciferri RR. A non-intrusive and reactive architecture to support real-time ETL processes in data warehousing environments. Heliyon. 2023;9(5).
25. Ren S, Wang T, Lu X. Dimensional modeling of medical data warehouse based on ontology. In: 2018 IEEE 3rd International Conference on Big Data Analysis (ICBDA). IEEE; 2018. p. 144-9.
26. Karami M, Rahimi A, Shahmirzadi AH. Clinical data warehouse: an effective tool to create intelligence in disease management. Health Care Manag (Frederick). 2017;36(4):380-4.
27. Grammatico-Guillon L, Shea K, Jafarzadeh SR, Camelo I, Maakaroun-Vermesse Z, Figueira M, et al. Antibiotic prescribing in outpatient children: a cohort from a clinical data warehouse. Clin Pediatr (Phila). 2019;58(6):681-90.
